# A new version of the HBSC Family Affluence Scale - FAS III: Scottish Qualitative Findings from the International FAS Development Study

**DOI:** 10.1007/s12187-015-9325-3

**Published:** 2015-08-02

**Authors:** Jane E. K. Hartley, Kate Levin, Candace Currie

**Affiliations:** Scottish Collaboration of Public Health Research and Policy, University of Edinburgh, Edinburgh, UK; NHS Greater Glasgow and Clyde, Glasgow, UK; CAHRU University of St Andrews, St Andrews, UK

**Keywords:** Health inequalities, Family affluence scale, Health behaviours, Qualitative research, Validation, Adolescents, Health indicators, Health behaviour in school-aged children (HBSC)

## Abstract

A critical review of the Family Affluence Scale (FAS) concluded that FAS II was no longer discriminatory within very rich or very poor countries, where a very high or a very low proportion of children were categorised as high FAS or low FAS respectively (Currie et al. 2008). The review concluded that a new version of FAS - FAS III - should be developed to take into account current trends in family consumption patterns across the European region, the US and Canada. In 2012, the FAS Development and Validation Study was conducted in eight countries - Denmark, Greenland, Italy, Norway, Poland, Romania, Slovakia and Scotland. This paper describes the Scottish qualitative findings from this study. The Scottish qualitative fieldwork comprising cognitive interviews and focus groups sampled from 11, 13 and 15 year-old participants from 18 of the most- and least- economically deprived schools. These qualitative results were used to inform the final FAS III recommendations.

## Introduction

In the WHO European region there are gross social and economic inequalities within and between countries. Health inequity accompanies these inequalities (Jakab and Marmot 2012), and it is one of the main priorities of public health research and practice to document, explain and design interventions to prevent and reduce social inequalities in health and health behaviours. International studies on health inequalities among adults are widely reported and the range of standard indicators used to measure social status include employment status, occupational class and educational level. Until recently, health inequalities among young people have been a relatively neglected field of research compared to health inequalities among adults. However there is a strong rationale for measuring and addressing health inequalities in adolescence. This is a life stage where many health behaviours are initiated, tracking into adulthood (Kelder et al. 1994). Furthermore, the social context of young people - and in particular the school context - make it amenable to interventions directed at modifying health behaviours and narrowing health inequalities (Department of Health 1998).

There are, however, difficulties in measuring socioeconomic status (SES) within a population still in full-time education and without occupational status; the socioeconomic status of parents is usually used as an indicator of adolescent SES. However, within survey collections cross-nationally, adolescent reporting of parental SES measures suffers from high rates of missingness (Currie et al. 2008). Although there is evidence that many young people do know their parents’ occupation and where they work (West et al. 2001; Vereecken and Vandegehuchte 2003), they do not always provide sufficient information for occupational coding, or indeed they may give no response. The lack of robust indicators of socioeconomic status may have hampered survey research in the area of health inequalities among young people until recently. However, in the last 10 to 15 years, with the development of new measures of SES, international research in the area has grown, and much of the cross-national comparative work has been conducted by the Health Behaviour in School-Aged Children: WHO Collaborative Cross-National Study (HBSC) and further reported by WHO (2013), UNICEF (2007) and OECD (2011).

The Health Behaviour in School-Aged Children (HBSC) Study originally developed a Family Affluence Scale (FAS) in 1998 to measure material affluence, a proxy for socioeconomic status measured by more traditional means of parental occupation or education (Currie et al. 1997). This measure overcame the problem of missingness common in the collection of parental occupation and educational status of parents; response rates are high for the component items. The FAS includes items which reflect the material resources that a family has, their patterns of consumption and their purchasing power in different countries across Europe and North America. Over time there has been a need to revise and update the FAS to keep it relevant to temporal changes in living conditions, societal norms and technology, and there have been two versions of the HBSC FAS to date (FAS I and FAS II) which have incorporated slightly different items. The use of FAS within HBSC has resulted in a large body of work describing the variation in a range of health outcomes and health related behaviours related to family affluence with effects at both individual and country level (Currie et al. 2008; ter Bogt et al. 2014; Elgar et al. 2013; Pickett et al. 2013; Levin et al. 2011, 2012). FAS has also been used and, in some cases, adapted by other studies around the world (Koivusilta et al. 2006; Liu et al. 2012; Park et al. 2012; Sleskova et al. 2006; Von Rueden et al. 2006; Williams et al. 1997). It has also been included in an Index of Child Well-being in the EU (Bradshaw et al. 2009) and as an item in UNICEF’s index of children’s material wellbeing in its comparative report of child well-being (UNICEF 2007).

A critical review of the FAS in the measurement of inequalities in health across Europe concluded that FAS II was no longer discriminatory within very rich or very poor countries, where a very high or a very low proportion of children were categorised as high FAS or low FAS respectively (Currie et al. 2008), and the conclusion was that a new version of FAS, FAS III, should be developed to take into account current trends in family consumption patterns across the European region, the US and Canada. This paper describes qualitative findings of a ‘FAS Development and Validation Study’.

## The FAS III Development and Validation Study (FAS III DVS)

In 2012/2013 the FAS III DVS was undertaken. This validation study aimed to develop a new and valid Family Affluence Scale (FAS III), comprising items that reflect market forces, economic trends, technological advances, as well as cultural, social and geographical norms in consumption across Europe and North America. To achieve geographic and socioeconomic diversity, albeit mainly within the developed economies, eight countries were selected to participate in this study: Denmark, Greenland, Italy, Norway, Poland, Romania, Scotland and Slovakia. The validation study sought to test a list of new candidate items for FAS III, and to check that items comprising FAS III enable segmentation of populations in these economically different countries. FAS III was required to provide a highly flexible way to compare absolute and relative inequalities between groups of children, both within and between countries. The ultimate aim was to develop a new version of FAS, FAS III, that would be relevant for the measurement of material affluence among young people across Europe and North America in 2012.

Specifically, the aim of this paper is threefold. First, to present the research methods of the FAS III Validation Study; second, to present detailed qualitative findings from one country, Scotland, that took part in the study; and third, to detail the findings from qualitative work in Scotland, which, in tandem with the FASIII DVS qualitative data from the other countries and the quantitative data (reported elsewhere), will inform this latest version of FAS (FAS III).

## Methods of the FAS III DVS

A mixed-method design was developed, combining qualitative and quantitative data. The design had the following core components:

1. a school-based pupil survey which tested new items of family affluence;
2. a school-based test-retest survey on a subset of the original pupil sample;
3. a parent survey on a subset of the pupil sample;
4. cognitive interviews with individual pupils who were surveyed;
5. focus group discussions with pupils who were surveyed on their perceptions of family affluence and wealth.

The data to be reported here are the *qualitative* data from *Scotland:* that is to say, the data generated from “4” and “5” above. A sub-sample of children was invited to participate in qualitative work to explore cultural differences, as well as issues of comprehension, item relevance, ethics and the sensitivity of the candidate FAS items. The protocol recommended ten cognitive interviews and ten focus groups per country. The Scottish data were collected in the autumn of 2012. This involved visiting 18 schools and sampling from the most- and the least-economically deprived schools, and from ages 11, 13 and 15 years (Table 1).

**Table 1:** FAS III Validation Study Scotland: qualitative study

Type of interview	Urban areas			Rural areas		
	Deprivation			Deprivation		
	High	Med	Low	High	Med	Low
Number of focus groups *per age group (years)*:	**1** *(11)* **1** *(15)*		**1** *(13)*	**3** *(11)* **1** *(13)* **1** *(15)*		**1** *(11)*
Number of individual interviews *per age group (years)*:	**1** *(13)* **1** *(15)*		**2** *(15)*	**3** *(11)* **1** *(13)* **1** *(15)*		**1** *(11)*

The fieldwork was undertaken by the first author

Project information sheets and opt-out consent forms were sent to the contact teacher in each school to pass on to the pupils’ teachers. On the day of the data collection, the project was explained in detail to all participants before they were invited to fill out consent forms, having read the information sheet. All names and place names in this report are pseudonyms in order to protect participant anonymity.

## Selection of Candidate Items for the FAS III Validation Study

The candidate items to be tested were selected as an outcome of international expert consultation across HBSC member countries in Europe and North America. The items were tested in eight countries, thereby providing a geographic, economic and cultural spread: Denmark, Greenland, Italy, Norway, Poland, Romania, Scotland and Slovakia. A form was sent to all HBSC member-country principal investigators requesting proposals for new candidate items to be included in FAS III, based on their knowledge and expertise about their country and the scientific area. In all, 19 of the 43 HBSC countries responded with proposed new items. Countries spanned Europe Canada and the US. A scan of the literature was also carried out and additional items were added to the list. Following discussions by the HBSC Social Inequalities Focus Group, an international scientific group from the network with expertise in the area, the list was shortened and sent out once more to all principal investigators for comment. This process produced the items to be included in the FAS III Validation Study (Table 2). The items are structured as ‘old’ used in FASII, ‘refined’—adapted FASII items, and ‘new’ items in FASIII.

**Table 2:** Main items selected for inclusion in the FAS III Validation Study, including ‘old’ items previously used in FASII, ‘refined’ FASII items, and ‘new’ FASIII items

Variable (history)	Item wording
C7comp (old): (C8car, old): (C9hol, old): (C10bedroom, old): (C20welloff, old): (C24hungry, old): (C21abroad, refined): (C25ownbed, refined): (C32owncomp, refined): (C22afford, new): (C23outdoor, new): (C26holhome, new): (C27dishwash, new): (C27washer, new): (C27dryer, new): (C28internet, new): (C29paywork, new): (C30money, new): (C31clothes, new): (C33bathroom, new): (C34ipod, new): .	How many computers (PCs, Macs or laptops) does your family own? Does your family own a car, van or truck? During the past 12 months, how many times did you travel away on holiday with your family?’ Do you have your own bedroom for yourself? *Children were asked to imagine that the ladder pictured shows how Scottish/Danish/*etc. *society is made up.[…]* Do you ever go to bed hungry because there is not enough money to buy food? How many times did you travel abroad for holiday/vacation last year? Do you have a bed of your own? Do you have your own computer? How many times in the last month have you not been able to afford to do something you wanted to do (e.g. go out with friends, do sports, buy clothes, go to a disco)? Does your home have an outdoor space attached, (e.g. garden)? Does your family have a holiday (vacation) house/apartment? Does your family have a dishwasher? Does your family have a washing machine? Does your family have a tumble dryer? Do you have internet access at home? Do your parents pay people to do work in your home (e.g. cleaning, cooking, gardening)? Do you receive pocket money? Do you wear clothes that belonged to others before you (secondhand clothes) or share clothes with your siblings? How many bathrooms (room with a bath or shower) are in your home? Do you have an Ipod or other personal music player?

### Focus Groups

All focus groups lasted between 30 and 50 min. The focus groups were carried out in a flexible way to respond to particular group dynamics and/or to the time available. The focus group exercise aimed to (a) determine young people’s understanding of potential items/question wording, and age-group differences in understanding, (b) highlight sensitive questions or items, and (c) reveal bias in response by SES, gender and/or geography. The key themes for every focus group were: *home/house* (car, bedroom, computer, bed, bath/shower room, white goods, internet access, garden, holiday home); *holidays/activities* (holidays, things can’t afford to do); *material possessions* (clothes, Ipod); *family life* (problem paying for things, going to bed hungry, people working for the family). In order to facilitate full participation and to protect participants’ privacy, a range of ‘activity sheets’ were designed. Each pupil was encouraged to respond to questions on the sheets without recourse to others in the group. A discussion then followed centred around the answers given.

### Cognitive Interviews

Individual cognitive interviews were held with one participant from each school class. These participants completed the same questionnaire as their classmates, but, whilst doing so, were asked to tell the researcher what they were thinking when they answered each of the questions. Participants were prompted with questions such as, ‘Why did you pick that answer?’ or ‘Can you explain more about your decision?’ This was a valuable technique that generated useful data. Due to both the number of items under examination and time constraints, some of the items were assessed by interview only. These were generally more objective items (for example, ‘Do you have your own computer?’), although in some cases interviews were a preferred method to gain a more in-depth understanding of the pupils’ response to the item (for example, in the case of item C20—see below).

### Analysis

The qualitative analysis was carried out in order to assess ways in which participants interpret and understand the items, investigating in particular the sensitivity and suitability of items.

## The Scottish Qualitative Results

To repeat, we are dealing here with only the qualitative analysis for Scotland, and we shall consider first the ‘refined’ items, and thereafter the ‘new’ items. Take the *refined* items. *C25ownbed. Do you have a bed of your own?* was clearly understood, and every participant reported having their own bed, therefore it would not differentiate affluence and so is not recommended, although it may have discriminatory power in developing countries. The item *C32owncomp. Do you have your own computer?* was generally well-understood, but it generated two common participant queries. The first was: did it ‘count’ if the computer was shared with a sibling or parent; and the second was did it ‘count’ if it was broken? This latter might be an especially relevant consideration during times of economic recession. Overall there was not enough detail from the data to judge whether this item was a valid indicator of family affluence from a qualitative perspective.

There were two refined items which referred to holidays. The first *C26holhome. Does your family have a holiday (vacation) house/apartment?* was discussed in cognitive interviews. The main matter of interpretation was what constituted ‘family’, and whether that included family holiday homes owned by extended family such as grandparents, aunties and uncles; and, related to this, whether or not visiting extended family in their main home counted as having a family holiday home. The second refined holiday-related item was *C21abroad. How many times did you travel abroad for holiday/vacation last year?* It was generally well-understood. There were, however, four matters arising from it. First, there was confusion over whether going to England counted as ‘abroad’. Second, often participants answered the question based on all of their holiday experiences, not just those from the last year. Third, sometimes participants described family holidays as accompanying their parents on a work-related trip to another country. Fourth, this item has the potential to be sensitive for those who have *not* been away on holiday. Overall, this item did appear to capture the difference in family affluence, but, given these considerations, it was decided that this should be re-worded as, ‘How many times did you and your family travel out of (name of country) for a holiday/vacation last year?’

The *new* items are now considered. With respect to item NewC20: *NEW C20welloff: children were asked to imagine that the ladder pictured shows how Scottish/Danish/*etc. *society is made up. At the top of the ladder are the people who are the best off. They have ‘the most money, the highest amount of schooling and the jobs that bring the most respect’. At the bottom of the ladder are the people who are the worst off. They have ‘little or no education’, ‘no job’, or ‘jobs that make little money’. Children were then asked to ‘think about your family. Please tell us where you think your family would be on this ladder. Fill in the circle that best represents where your family would be on this ladder (scale from 0 to 10)’.* This item generated a range of responses. The first was the matter of relative status. When the participant is answering the question, we cannot know what their reference group is: that is, their community; those whom they see on television; their friends at school. The qualitative data made it clear that the participants referred to a variety of groups, and for this reason the subjective perception of wealth is not comparable to objective measures of actual wealth. Take Beth (*Sch6IIGirlP7LowDeprivation*), for example: her estimation of her family wealth was: ‘And we’re not, like, poor, but we have enough money, but we don’t have, like, millions of pounds.’ Her father had a ‘good’ job and her mother was a trainee teacher. She wavered between a ‘seven’ and an ‘eight’ rating on the scale.

The item also highlighted the conceptual complexity of the construct ‘family’: is it their nuclear family; their extended family; or, if their parents are separated, which family is being rated? The qualitative data showed that participants often selected that segment of their family whom they considered to be the ‘best off’. For example, when Matthew (*Sch8IIBoyP7HighDeprivation*) was asked whom he included when he rated his family, he opted for his extended family because he felt that their jobs would increase his family’s score. His rating was a ‘seven’. For Matthew, his family comprised his mother (a nurse), his grandfather (who had a breakdown recovery business), his father (a farmer), his uncle Barry (who designed sheds), his auntie Jacqui (who was a holiday-let house-keeper), and his auntie Julia and uncle Peter (who worked in a carpentry business). Matthew thought his father’s occupation would keep the rating at a ‘six’, but by including his uncle Barry it would rise to a ‘seven’ because not only did he design sheds, he had ‘quite a big house as well’, and ‘he’s quite rich’ [Laughter]. Matthew’s mother, a nurse, also attracted a ‘seven’.

This item is also a sensitive one, and raises ethical issues. Especially for the younger participants, the impression they often gave was that this was the first time they had been asked to consider ‘rating’ their family. The FAS validation process in general was extremely sensitive for those who were most deprived, but this item in particular forced a consideration of a comparison which hitherto they might not have made. Even so, the power of relativity appeared to work at times as a buffer, even for the most deprived participants. Ryan (*Sch13IIBoyS4HighDeprivation*) compared his family to those wherein there was drug addiction:

I’d say zero were drug addicts and that. People that all they do is try to get money to get their fix. Then, when you go up, it’s just less fortunate people. I’d say I was eight because nobody in my family are drug addicts; we’ve all just got what we need. There’s no family rows with other families.

We turn here to item *NewC22afford. How many times in the last month have you not been able to afford to do something you wanted to do (for example, go out with friends, do sports, buy clothes, go to a disco)?* This item was discussed both in the cognitive interviews and in the focus-group discussions. Four issues emerged. The first was that participants tended not to focus on the ‘affording’ part of the question. Take Jake’s (*Sch11IIBoyP7HighDeprivation*) responses:

I: And how many times in the last month have you not been able to afford to do something you wanted to?

Jake: I’d, probably, just say once.

I: Okay, can you think of what it was?

Jake: It was going to this Climbing Centre. It’s, you get, you pay two pound for the shoes, and then you pay, like, 20 pound just to get a card for a couple of hours.

I: Yeah.

Jake: Yeah, but, like, the card’s for the whole day, so, it’s good.

I: Okay, okay.

Jake: But we normally end up going at night, because my dad works, and then he tries to get away.

I: Okay, but this time in the last month it was a time that you didn’t have the money for it?

Jake: No, I went with my dad but not my friends.

Here, therefore, is an example of a response which had more to do with a non-financial reason.

Secondly—and still with the ambiguity which attached to the concept of ‘being able to afford’ something—there were a few occasions when participants assumed they would not be allowed to go, possibly because of monetary restrictions, and for that reason they did not even ask their parents; they had an awareness of the financial constraints within which their parents lived. Beth (*Sch6IIGirlP7LowDeprivation*) was reluctant to ask her mother for money for certain things because she knew her mother could not afford it, even though her mother would not admit it. Beth explained: ‘Well, but, usually, mum doesn’t say I can’t afford it. She says, ’I don’t really want to go there, because it’s too babyish’, or something. […] Well, she doesn’t say it’s too babyish she just says, ‘No, I don’t think so, because we should do this’. ‘And I’m, like, okay, right, I’ll do this.’ The third issue was that this item did not address the family’s daily financial status. Often participants would cite the things they could not afford, such as a private jet, a tropical holiday, or other expensive leisure pursuits, rather than day-to-day things that would give a more accurate reflection of the family’s daily financial status. Other participants found it difficult to imagine real things they could not have. The final matter arising from this item was an ethical one. This is a potentially sensitive item for those who have experienced not being able to afford something, as Ryan’s (*Sch13IIBoyS4HighDeprivation*) comments suggest. Ryan stated that he had been unable to afford things in the last month, but then added the slightly defensive caveat that he could have afforded them, but that he ‘just never had it at the time’, which implies that he felt the need to justify his inability to afford things.

The next new item *(NEW C23outdoor. Does your home have an outdoor space attached, (*e.g. *garden)* was discussed in cognitive interviews. It was a generally well-understood item, with no apparent comprehension, ethical or sensitivity concerns. But there is a proviso. An ‘outdoor space’ could range from the two-acre garden of a large mansion to a communal garden at the bottom of a high-rise apartment block. This definitional imprecision would warrant clarification for it to be included in the FAS III main study.

New items C27,28, 33 and 34 were all considered in the cognitive interviews and can be dealt with briefly; C29 and 31 require further consideration. Briefly, *NewC27dishwash. At home do you have the following: Dishwasher?, NewC27dryer. At home do you have the following: Tumble dryer?, NewC27washer. At home do you have the following: Washing machine?* were unproblematic and therefore could be recommended as items to be considered for FASIII. *NewC28internet. Do you have Internet access at home?* was also generally unproblematic, bar the fact that all participants appeared to have internet access; therefore this item would not discriminate between groups. Similarly, *NewC33bathroom. How many bathrooms (room with a bath or shower) are in your home?* generated no concerns. As for *NewC34ipod. Do you have an iPod or other personal music player?,* it prompted three matters. The first is the use of the wording ‘iPod’: does it mean only an *Apple* brand of mp3 player, or should this be re-worded as ‘mp3 player or other personal music player’? The second issue is that these categories of technology very quickly become obsolete. Already the smartphone has, in many cases, replaced the iPod, and this too will be replaced by new technology. The third issue is a methodological issue. Many participants queried whether or not the item was asking specifically about their own ownership, or did it ‘count’ if there was an iPod or other personal music player that they shared with a sibling or parent? This item, therefore, is not recommended. Although it could be updated with different wording, the matters of technological obsolescence and ownership would remain problematic.

Unlike the foregoing items C27,28, 33 and 34, items NewC29 and NewC31 merit further consideration. Take *NewC29paywork. Do your parents pay people to do work in your home (*e.g. *cleaning, cooking, gardening)?* This item was discussed both in the cognitive interviews and in the focus group discussions. A number of concerns emerged, the first of which was that some participants did not take the item ‘seriously’, presumably because they thought that having paid help in the home seemed like such a fanciful and unlikely notion. A focus-group discussion with some girls (*Sch11FGGirlsP7HighDeprivation*) drew a distinction between realistic paid help, such as caring for an elderly relative, or having windows cleaned, and the somewhat unrealistic presence of a ‘maid’, a ‘butler’, a ‘bouncer’ or a ‘chauffeur’. The intended meaning in the item is that paying people to work in the home is on-going, not a one-off event, but participants cited ad hoc work, such as a builder coming to fix the roof, or someone to landscape the garden. The inference to be drawn from these responses is that the item does not indicate the family’s affluence. And, for some participants, like Ross (S*ch8FGMixedP7HighDeprivation*), paid work included doing chores in exchange for pocket money: ‘Mum and Dad pay for me and my brother to wash the dishes; we get a tenner a month’. Finally, there is an ethical consideration: it was apparent that having paid home help was a social-class signifier. For example, in one focus group, upon learning to her surprise that her ‘best friend’ Ruth had a cleaner and a babysitter at home, Jenny (*Sch3FGGirlsS2HighDeprivation*) said in ‘jest’ that she did not want to be friends with her anymore. An interpretation of this exchange is that Jenny became aware of a hitherto hidden social-status signifier which she felt separated her from Ruth as it implied a different state of affluence. The item did appear to be a valid socio-economic discriminator, but could be refined along the lines of ‘Do your parents pay people from outside the family to work at your home on a regular (that is, on a daily or weekly) basis?’

Like item NewC29, item *NewC31clothes: Do you wear clothes that belonged to others before you (second-hand clothes) or share clothes with your siblings?* generated a range of issues in the cognitive interviews and the focus-group discussions. To be sure, this item seemed to stir the emotions, attracting a variety of very lively responses, even somewhat *risque* hilarity and teasing. The weight of opinion seemed to show a preference for shared rather than second-hand clothes, especially within a family. Ruth (*Sch3FGGirlsS2HighDeprivation*), for example, stated: ‘I just think of sharing clothes with my mum, because I’ve just kind of reached the same sort of size as her, so usually share clothes with my mum for things.’ But another girl from the same focus group, Anna, pointed out that siblings sometimes were disinclined to share their clothes. As for sharing clothes with school friends, the opinion was mixed. Scott (*Sch6FGMxdP7LowDeprivation*) stated that ‘when someone comes round to my house or we go out to play football and they come back and you get really muddy and they didn’t bring a spare change of clothes, I’ll give them some of my clothes.’ But then, to the amusement of the others, he wavered: ‘I don’t really know…because they’re my clothes and my privacy and I don’t want any person’s germs’, a view echoed by Jimmy (*Sch13FGBoysS4HighDeprivation*) who thought the wearing of a friend’s clothes felt ‘weird’ and ‘sweaty’. The girls were less disinclined either to share clothes, or to have second-hand clothes, provided they were ‘cool’ and ‘vintage’. On balance, this item may have benefited from having discrete sub-categories for swapping, choosing to share, having to share, and wearing second-hand clothes by choice? However, because ‘second-hand clothing’ was not separate from ‘shared clothing’ in the questionnaire it cannot be recommended as a new FAS item.

## Summary of the Recommendations

Based on the foregoing analysis of the qualitative data, Table 3 indicates the recommendations (column 3) derived from the FAS III Development and Validation Study. Only the ‘refined’ and ‘new’ items, as indicated in Table 2, are referred to. These recommendations are subject to consideration with the corresponding FASIII quantitative data.

**Table 3:** FAS III Development and Validation Study: recommendations

Variable (history)	FAS III Item wording	Recommendation: FAS III DVS
(C21abroad, refined): (C25ownbed, refined): (C32owncomp, refined): (C22afford, new): (C23outdoor, new): (C26holhome, new): (C27dishwash, new): (C27washer, new): (C27dryer, new): (C28internet, new): (C29paywork, new): (C30money, new): (C31clothes, new): (C33bathroom, new): (C34ipod, new): .	How many times did you travel abroad for holiday/vacation last year? Do you have a bed of your own? Do you have your own computer? How many times in the last month have you not been able to afford to do something you wanted to do (e.g. go out with friends, do sports, buy clothes, go to a disco)? Does your home have an outdoor space attached, (e.g. garden)? Does your family have a holiday (vacation) house/apartment? Does your family have a dishwasher? Does your family have a washing machine? Does your family have a tumble dryer? Do you have internet access at home? Do your parents pay people to do work in your home (e.g. cleaning, cooking, gardening)? Do you receive pocket money? Do you wear clothes that belonged to others before you (secondhand clothes) or share clothes with your siblings? How many bathrooms (room with a bath or shower) are in your home? Do you have an Ipod or other personal music player?	Item to be retained and re-worded as: ‘How many times did you and your family travel out of (name of country) abroad for holiday/vacation last year?’ Item to be rejected. Insufficient data to judge its validity. Item to be rejected. Insufficient data to judge its validity. Item to be rejected. The term ‘outdoor space’ needs greater clarity and the item requires subsequent piloting. Item to be rejected. Item to be rejected. Item to be retained Item to be retained Item to be retained Item to be retained Item to be retained and re-worded as: ‘Do your parents pay people from outside the family to work at your home on a regular (that is, on a daily or weekly) basis?’ Item to be retained. Item to be rejected Item to be retained Item to be rejected.

## Conclusion

The FAS must take account of the economic, technological and social changes which are afoot, and be refined accordingly. The qualitative study reported here has been part of that endeavour. The purpose here has been to refine the HBSC Family Affluence Scale (FAS), a measure of material affluence which can be administered to young people who are not in a paid occupation. The scale does not rely upon the need for accurate information about parental education and occupation. The findings reported here relate only to the Scottish data. Comparable qualitative studies were also conducted in each of the other countries participating in the FAS III DVS - Denmark, Greenland, Italy, Norway, Poland, Romania and Slovakia. A recurring theme has been to be mindful of constructs which no longer are as fixed in their meaning as they used to be; that of ‘family’ is an example. The study here has attempted to gain some understanding of how these young participants are influenced by their own lived experiences when they responded to items in the survey, and to try to discern the meanings which they intended by their responses. By refining the wording in some items, or even rejecting items, the recommendations list in Table 3 have sought to minimise ambiguity and subjectivity whenever possible, and to maximise the validity and relevance of the items. Apart from dealing with these matters of interpretation and conceptual precision, the recommendations have also been very careful to attend to the sensitivities of these young people, particularly with reference to those items which might reinforce or prompt a fuller awareness of poverty and deprivation. The selected items were next included in a pilot survey in each of the countries participating in the FAS III DVS study. The purpose of the survey was to assess the scale properties of the new items when combined as a single scale –FASIII across the different social, economic and cultural contexts of the countries. The findings of this study are the focus of a separate paper and are currently presented elsewhere (Anonymised for review 2013). All candidate items will be included in the 2013/2014 HBSC cross-national survey in Europe and North America as part of the FASIII.
